# A novel 9-gene signature for the prediction of postoperative recurrence in stage II/III colorectal cancer

**DOI:** 10.3389/fgene.2022.1097234

**Published:** 2023-01-10

**Authors:** Cheng Xin, Yi Lai, Liqiang Ji, Ye Wang, Shihao Li, Liqiang Hao, Wei Zhang, Ronggui Meng, Jun Xu, Yonggang Hong, Zheng Lou

**Affiliations:** ^1^ Department of Colorectal Surgery, Changhai Hospital, Shanghai, China; ^2^ Department of Head and Neck Surgery, Renji Hospital, School of Medicine, Shanghai Jiaotong University, Shanghai, China; ^3^ Department of Gastrointestinal Surgery, Changhai Hospital, Shanghai, China

**Keywords:** stage II/III colorectal cancer, risk score, recurrence-free survival, prognostic signature, immune infiltration

## Abstract

**Background:** Individualized recurrence risk prediction in patients with stage II/III colorectal cancer (CRC) is crucial for making postoperative treatment decisions. However, there is still a lack of effective approaches for identifying patients with stage II and III CRC at a high risk of recurrence. In this study, we aimed to establish a credible gene model for improving the risk assessment of patients with stage II/III CRC.

**Methods:** Recurrence-free survival (RFS)-related genes were screened using Univariate Cox regression analysis in GSE17538, GSE39582, and GSE161158 cohorts. Common prognostic genes were identified by Venn diagram and subsequently subjected to least absolute shrinkage and selection operator (LASSO) regression analysis and multivariate Cox regression analysis for signature construction. Kaplan-Meier (K-M), calibration, and receiver operating characteristic (ROC) curves were used to assess the predictive accuracy and superiority of our risk model. Single-sample gene set enrichment analysis (ssGSEA) was employed to investigate the relationship between the infiltrative abundances of immune cells and risk scores. Genes significantly associated with the risk scores were identified to explore the biological implications of the 9-gene signature.

**Results:** Survival analysis identified 347 RFS-related genes. Using these genes, a 9-gene signature was constructed, which was composed of MRPL41, FGD3, RBM38, SPINK1, DKK1, GAL3ST4, INHBB, CTB-113P19.1, and FAM214B. K-M curves verified the survival differences between the low- and high-risk groups classified by the 9-gene signature. The area under the curve (AUC) values of this signature were close to or no less than the previously reported prognostic signatures and clinical factors, suggesting that this model could provide improved RFS prediction. The ssGSEA algorithm estimated that eight immune cells, including regulatory T cells, were aberrantly infiltrated in the high-risk group. Furthermore, the signature was associated with multiple oncogenic pathways, including cell adhesion and angiogenesis.

**Conclusion:** A novel RFS prediction model for patients with stage II/III CRC was constructed using multicohort validation. The proposed signature may help clinicians better manage patients with stage II/III CRC.

## Introduction

Colorectal cancer (CRC) is a common and fatal gastrointestinal malignant tumor, with an estimated 147,950 new cases in 2020 (Siegel et al., 2020). Although great advances have been achieved in the perioperative management of CRC patients, a high postoperative recurrence rate remains a challenge that hampers remarkable improvement in patient outcomes (Xie et al., 2021). Most patients with CRC are diagnosed at stage II/III and present with resectable tumors (Brenner et al., 2014). After radical resection and subsequent adjuvant chemotherapy, 30%–60% of patients relapse within 5 years, with a dismal prognosis (Manfredi et al., 2006). Moreover, the individual benefits of adjuvant therapy are still questionable, resulting in potential over-treatment (Gray et al., 2007; Schippinger et al., 2007; Varghese, 2015). Therefore, accurate recurrence risk stratification and personalized adjuvant chemotherapy are of great significance for the long-term survival of patients with CRC.

To date, the most commonly used clinicopathologic factor for identifying high-risk stage II/III patients is using the tumor-node-metastasis (TNM) system. However, drug responses and clinical outcomes can vary widely in patients with CRC at the same TNM stage because of high genetic and epigenetic heterogeneity (Weiser et al., 2011; Ji et al., 2018; Lahoz et al., 2022). This clinical challenge indicates that the conventional TNM staging system is inadequate for risk evaluation, highlighting the urgent need to exploit novel and reliable molecular classifiers. With recent advances in high-throughput techniques, risk assessment and prognostic prediction have been dramatically improved by the use of gene expression profiling and bioinformatics technology (Koncina et al., 2020; Ahluwalia et al., 2021; Ghafouri-Fard et al., 2021). For stage II/III CRC, several prognostic signatures with predictive capacity for recurrence risk have been established by analyzing key cancer-associated pathways, such as autophagy and the tumor microenvironment (Mo et al., 2019; Zhang et al., 2021; Ren et al., 2022; Zhang et al., 2022). All of these models exhibit favorable predictive performance, but their credibility remains to be improved because they are mostly derived from a single GSE39582 dataset and lack multi-cohort and cross-platform validation.

In the current study, we explored genes with the potential to predict CRC recurrence and established a prognostic 9-gene signature with cross-cohort compatibility. The proposed model showed elevated accuracy and efficiency compared with previous models and clinical parameters for risk assessment. Moreover, this signature is closely related to multiple oncogenic pathways such as cell adhesion and angiogenesis. These findings might be meaningful in guiding postoperative prognostic stratification and in understanding the recurrence mechanisms of patients with stage II/III CRC.

## Methods

### CRC cohorts

Five independent CRC cohorts with TNM stage information were collected for survival analysis in the current study. Gene expression profiles and clinical data were obtained from the Gene Expression Omnibus (GEO) database. Among these cohorts, the GSE39582 cohort (N = 464) was used for training, while the GSE17536 (N = 110), GSE17538 (N = 142), GSE37892 (N = 129), and GSE161158 (N = 151) cohorts were used for validation. To ensure accuracy, samples with RFS <30 days were excluded from the subsequent analyses. In addition, 54 pairs of tumor and adjacent normal specimens were gained from patients who were diagnosed with stage II/III CRC and underwent surgical treatments at the Department of Colorectal Surgery at Shanghai Changhai Hospital. None of the patients received any local or systemic treatment before the surgery. Written informed consent for the use of clinical samples in medical research was obtained from all patients. All clinical procedures were approved by the Ethics Committee of Shanghai Changhai Hospital.

### Identification and functional annotation of RFS-associated genes

Three cohorts with the largest number of CRC samples (GSE17538, GSE39582, and GSE161158) were selected to screen reliable genes indicative of RFS. Univariate Cox regression analysis was conducted using the ‘survival’ package in the R environment (version 3.5.2) to screen for prognostic genes (*p* < .05) in three independent cohorts. The Venn diagram (Bardou et al., 2014) was subsequently used to screen for common genes with the ability to predict RFS in these three cohorts. The identified genes were then sent for Gene Ontology (GO) analysis for functional annotation on the Database for Annotation, Visualization, and Integrated Discovery (DAVID) online website (Sherman et al., 2022).

### Development and assessment of the risk signature

Using the commonly prognostic genes screened above, least absolute shrinkage and selection operator (LASSO) regression analysis based on the “glmnet” R package combined with multivariate Cox regression analysis based on the “survival” R package were performed to generate an optimal prognostic signature. The risk score was calculated as follows: Risk score = (coefficient 1 × expression level of gene 1) + (coefficient 2 × expression level of gene 2) + ... + (coefficient n × expression level of gene n). Each patient was assigned a risk score, and patients were classified into low- and high-risk groups according to the medium value of the risk scores. Kaplan–Meier (K-M) survival curves plotted by the “survminer” R package, calibration curves plotted by the ‘rms’ R package, and time-dependent receiver operating characteristic (ROC) curves plotted by the “timeROC” package were utilized to assess the prognostic ability of this signature. The area under the curve (AUC) values calculated using the K-M ROC R package were used to compare the predictive performance of our signature with clinical factors and two previously reported prognostic signatures that predicted postoperative recurrence in stage II/III CRC patients (Cheng et al., 2018; Zhang et al., 2022). Additionally, univariate Cox regression analyses were conducted to identify independent survival indicators in each cohort.

### Estimation of infiltrative abundances of immune cells

The single-sample gene set enrichment analysis (ssGSEA) method was conducted by the “GSVA” package to estimate the immune infiltration values of 23 immune cells in CRC samples. The relationships between immune infiltrative levels and risk scores were determined using Pearson correlation analysis.

### Functional analyses of the risk signature

To clarify the close association between the risk signature and patient prognosis, Pearson correlation analysis was performed to identify genes correlated with (*p* < .05) risk scores in the GSE39582 cohort. Based on the correlation coefficients, the top 1000 genes with positive or negative correlations were subjected to Gene Ontology-Biological Process (GO-BP) annotation and Kyoto Encyclopedia of Genes and Genomes (KEGG) enrichment using the DAVID online website. The enriched items and corresponding *p*-values were visualized by the “ggplot2” package.

### Immunohistochemical (IHC) staining

IHC assays were performed as previously described (Chen and Zhang, 2018). Sections were incubated with specific primary antibodies against MRPL41 (1:200 dilution, ab121821, Abcam, Cambridge, United Kingdom) and RBM38 (1:50 dilution, ab200403, Abcam, Cambridge, United Kingdom) at 4°C overnight. After incubation with the appropriate secondary antibodies at room temperature for 1 h, sections were stained with diaminobenzidine and hematoxylin. IHC scores were quantified as previously described (Chen and Zhang, 2018).

### Statistical analysis

Statistical analyses were performed using GraphPad Prism 6 software. Parametric data were analyzed using the Student’s t-test. Statistical log-rank *p* < .05 was considered significant.

## Results

### Construction of prognostic signature using RFS-related genes

**FIGURE 1:**
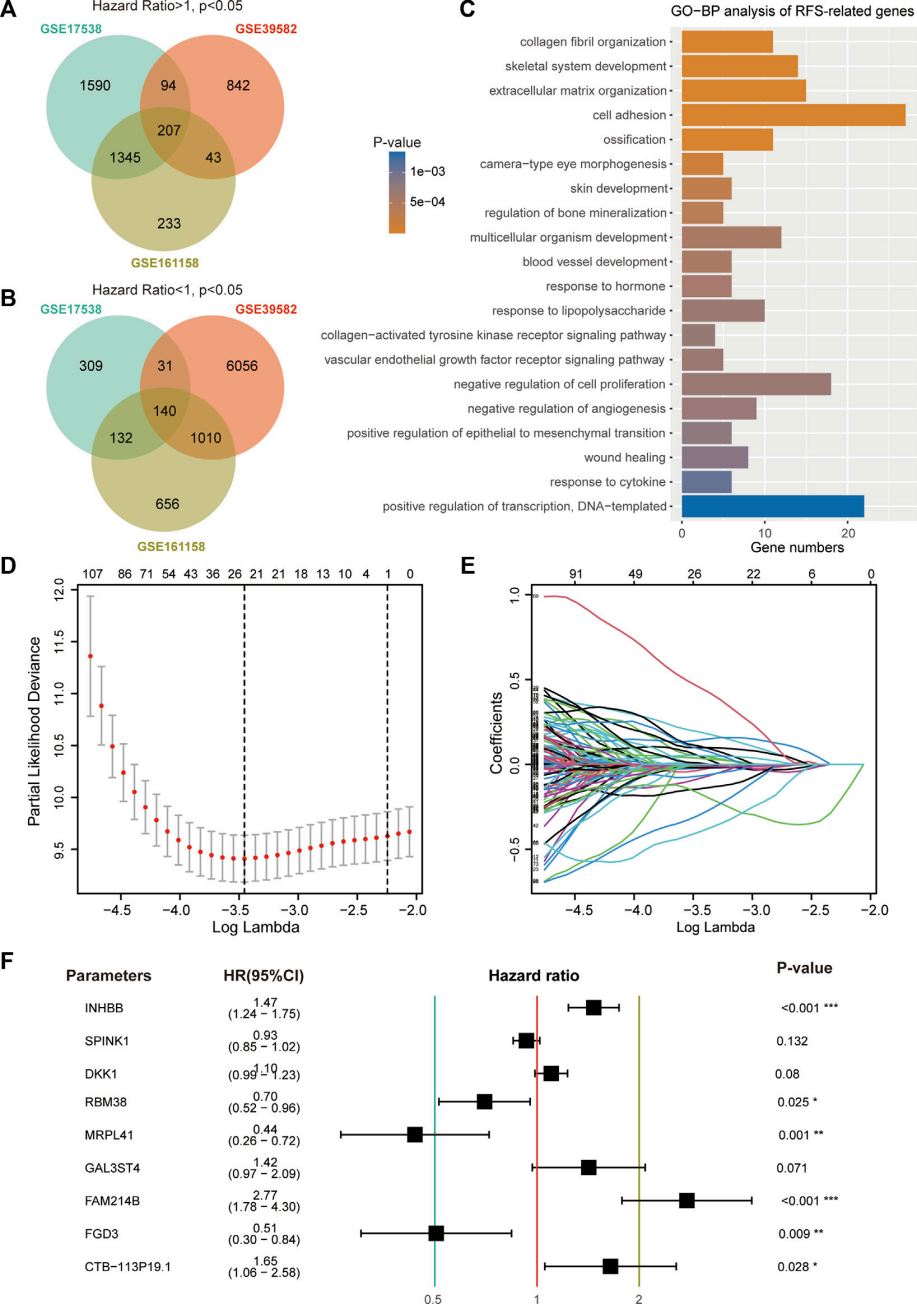
Construction of prognostic signature using RFS-related genes. **(A)** Venn diagram screened 207 prognostic genes with a hazard ratio >1 in three CRC cohorts. **(B)** Venn diagram screened 140 prognostic genes with a hazard ratio <1. **(C)** Top 20 enriched GO-BP terms of 347 RFS-related genes. **(D)** Cross-validation for tuning parameter (lambda) screening in the LASSO regression model. **(E)** LASSO coefficients of 24 RFS-related genes. **(F)** Hazard ratio, 95% CI, and *p*-value of the nine genes.

A total of 207 common genes with an HR > 1 (Figure 1A) and 140 common genes with an HR < 1 (Figure 1B) were selected for model construction. GO-BP analysis demonstrated that these 347 genes were closely related to functions of cell adhesion, proliferation, and angiogenesis (Figure 1C). LASSO regression analysis of these prognostic genes identified 24 candidate genes for subsequent analysis (Figures 1D, E). To avoid overfitting, multivariate Cox regression analysis was performed on these 24 genes to generate a 9-gene signature (Figure 1F). The risk score formula was developed as follows: Risk score = −0.82604 × expression level of MRPL41–0.68172 × expression level of FGD3—0.35399 × expression level of RBM38–0.06949 × expression level of SPINK1 + 0.09875 × expression level of DKK1 + 0.35278 × expression level of GAL3ST4 + 0.38681 × expression level of INHBB +0.50069 × expression level of CTB-113P19.1 + 1.01899 × expression level of FAM214B.

### Assessment of prognostic performance in training and validation cohorts

**FIGURE 2:**
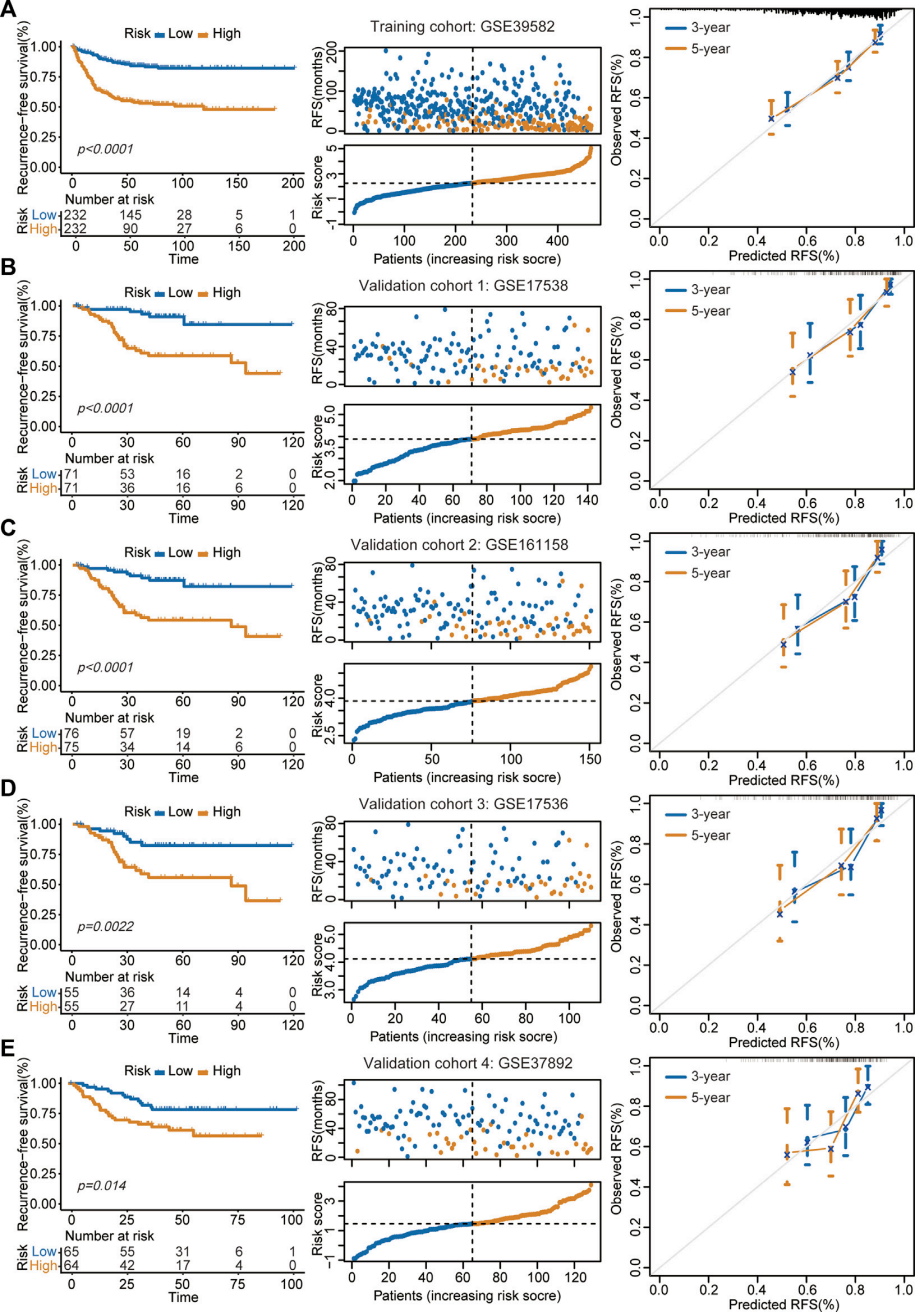
Assessment of prognostic performance in training and validation cohorts. **(A–E)** The proposed signature precisely captured the survival differences between two risk groups in GSE39582 **(A)**, GSE17538 **(B)**, GSE161158 **(C)**, GSE17536 **(D)**, and GSE37892 **(E)** cohorts, respectively. Left panel: K-M curves estimated the RFS differences between two risk groups. Middle panel: From top to bottom was the distribution of survival status and risk scores. The orange dots represented recurrence while the blue dots represented non-recurrence. Right panel: Calibration curves for the 9-gene signature.

In the GSE39582 cohort, the K-M survival curve showed a significantly decreased RFS time in patients in the high-risk group (Figure 2A, left panel). Patients in the high-risk group had markedly higher recurrence rates than those in the low-risk group (Figure 2A, middle panel). The calibration curve analysis showed that the survival probabilities predicted by our model were in good agreement with the actual survival probabilities (Figure 2A, right panel). In addition to the training cohort, the proposed signature also precisely estimated the different survival times and events of low- and high-risk patients in the GSE17538 (Figure 2B), GSE161158 (Figure 2C), GSE17536 (Figure 2D), and GSE37892 (Figure 2E) cohorts.

### Comparison of predictive accuracy between our signature and previous signatures

**FIGURE 3:**
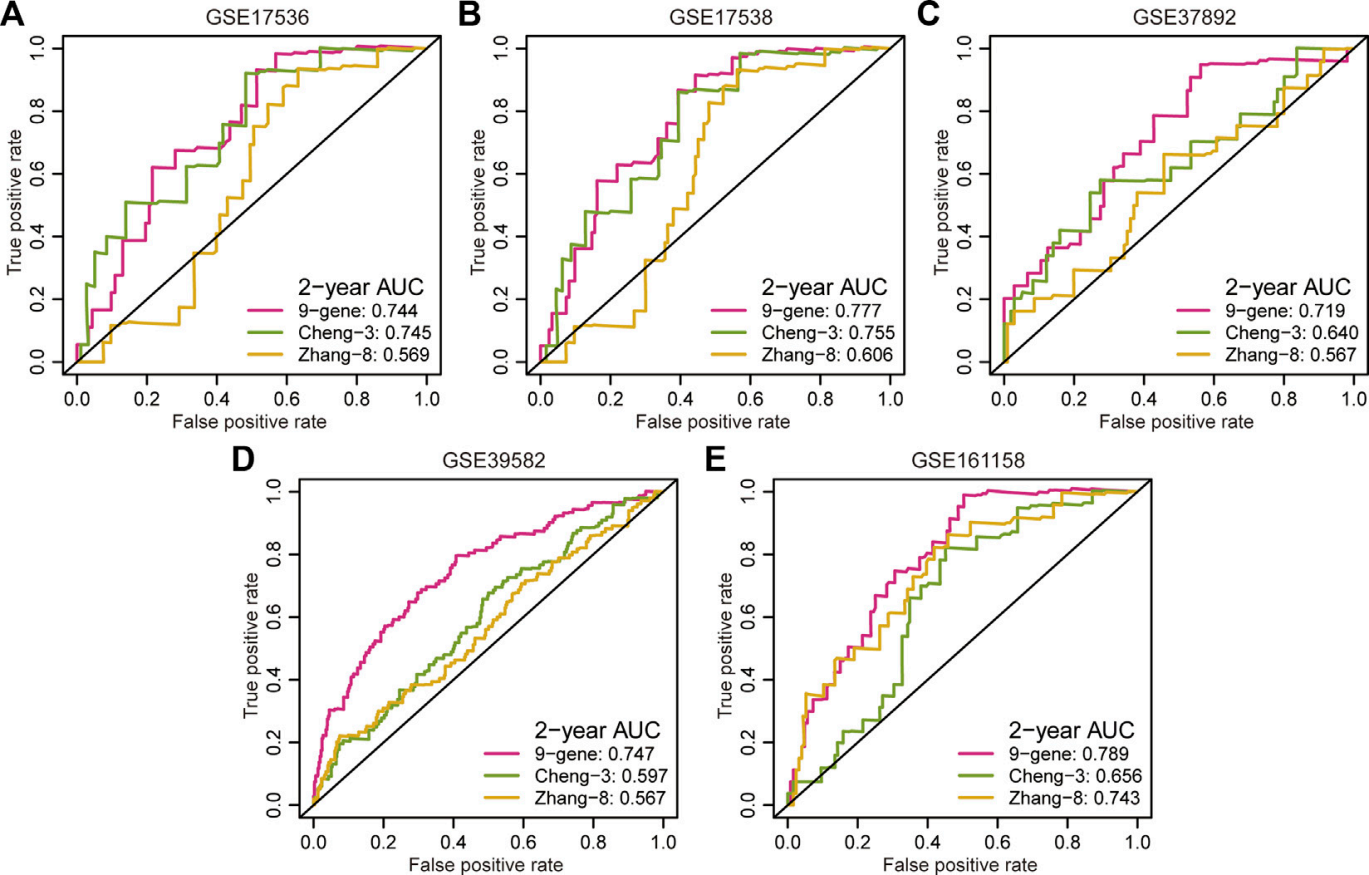
Comparison of predictive accuracy between our signature and previous signatures. **(A–E)** ROC curves investigated the predictive accuracy of gene signatures for 2 year RFS prediction in GSE17536 **(A)**, GSE17538 **(B)**, GSE37892 **(C)**, GSE39582 **(D)**, and GSE161158 **(E)** cohorts, respectively.

Multiple prognostic signatures for stage II/III CRC recurrence risk stratification were established, and we wondered whether our signature outperformed the published signatures in risk prediction. An AUC value analysis was adopted, and a high AUC value indicated high predictive accuracy. In addition to the second highest predictive accuracy in the GSE17536 cohort (AUC = 0.744, Figure 3A), the proposed signature showed the strongest prediction precision for 2 year RFS in the GSE17538 cohort (AUC = 0.777, Figure 3B), GSE37892 cohort (AUC = 0.719, Figure 3C), GSE39582 cohort (AUC = 0.747, Figure 3D), and GSE161158 cohort (AUC = 0.789, Figure 3E).

### Superiority evaluation of the 9-gene signature compared with clinical factors

**FIGURE 4:**
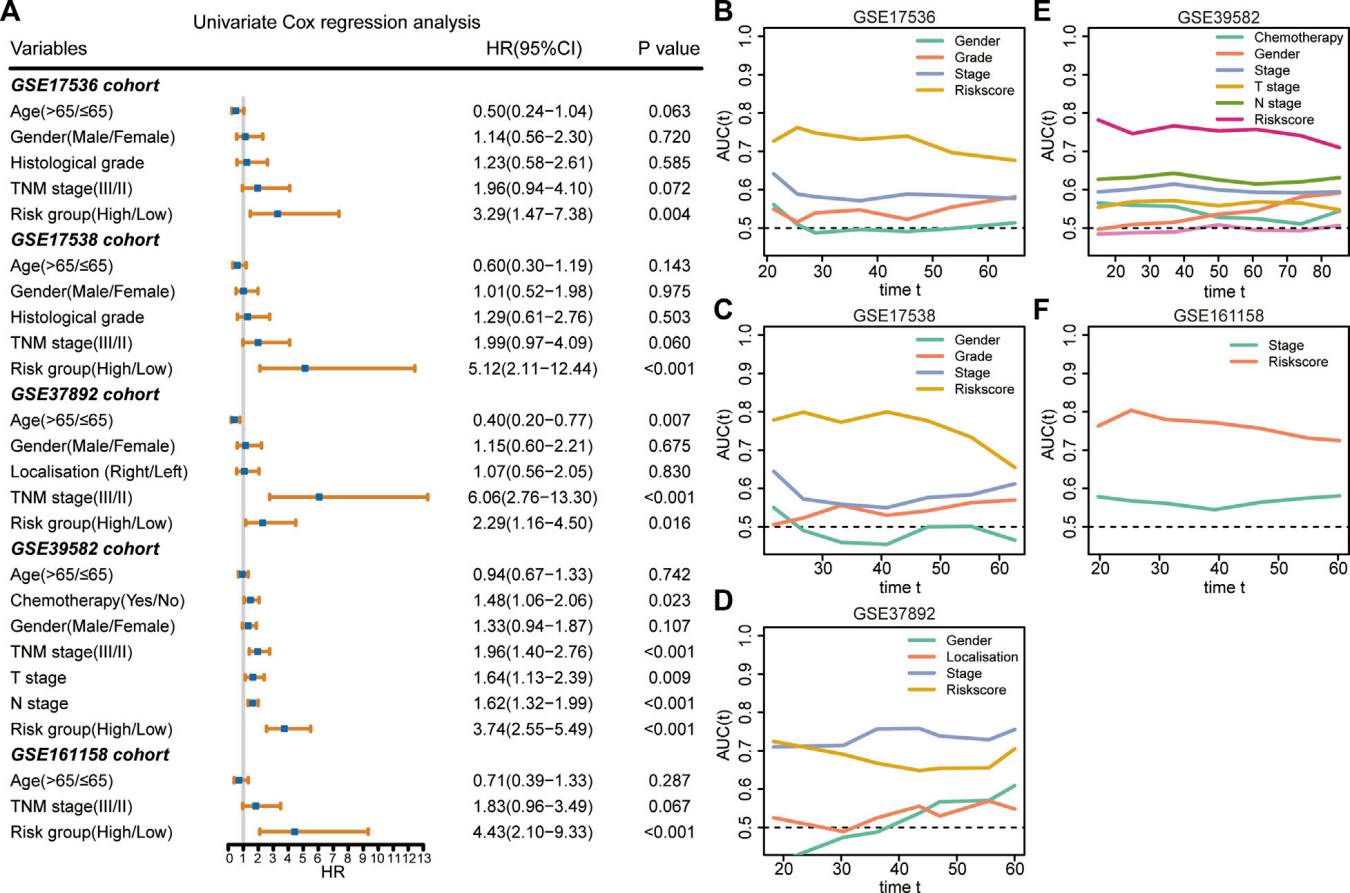
Superiority evaluation of the 9-gene signature compared with clinical factors **(A)** Univariate Cox regression analyses identified independent prognostic factors in each cohort. **(B–F)** Time-dependent AUC values of prognostic factors in GSE17536 **(B)**, GSE17538 **(C)**, GSE37892 **(D)**, GSE39582 **(E)**, and GSE161158 **(F)** cohorts, respectively.

The results of the univariate Cox regression analysis demonstrated that the 9-gene signature was associated with unfavorable survival in five independent cohorts (Figure 4A). To investigate the superiority of our signature in predicting RFS, time-dependent ROC analyses were performed to assess the accuracy of each predictor. The 9-gene signature exhibited higher dynamic AUC values than the clinical predictors over time in all the GSE17536 (Figure 4B), GSE17538 (Figure 4C), GSE39582 (Figure 4E), and GSE161158 cohorts (Figure 4F), except for the GSE37892 cohort (Figure 4D). These findings suggest that our signature outperformed clinical indicators for recurrence prediction.

### Differences in immune infiltration between two risk groups

**FIGURE 5:**
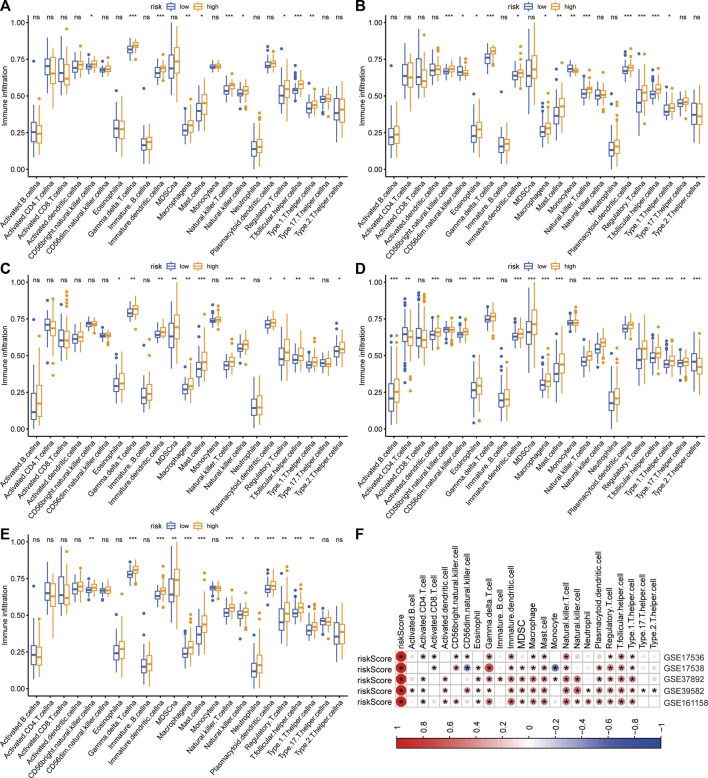
Differences in immune infiltration between two risk groups. **(A–E)** The immune infiltrative differences between two risk groups in GSE17536 **(A)**, GSE17538 **(B)**, GSE37892 **(C)**, GSE39582 **(D)**, and GSE161158 **(E)** cohorts, respectively. **(F)** The heatmap of correlations between immune infiltration and risk signature in each cohort. The blue color indicated negatively related and the red indicated positively related. *p < .05.

The immune infiltrative differences between the low- and high-risk groups were determined by ssGSEA analysis in each cohort (Figures 5A–E). The intersecting differences with the same alternative trends in these five cohorts were regarded as signature-related immunological changes. The results showed that eight immune cells, including γδ T cells, immature dendritic cells, macrophages, mast cells, natural killer T cells, regulatory T cells, T follicular helper cells, and Type 1 T helper cells, were upregulated in the high-risk group. Correlation analyses illustrated that in the GSE17536 and GSE17538 cohorts, the risk score was negatively associated with the infiltrative level of activated CD8^+^ T cells, whereas it was positively associated with the infiltrative level of regulatory T cells in all five cohorts (Figure 5F). We then investigated the association between the risk score and several immune checkpoints, such as CD274, CTLA4 and IDO1. As shown in supplementary Figures 1A–E, a substantial increase in the expression of HAVCR2 was found in the high-risk group in each CRC cohort. In addition, a significant decrease in the expression of GZMB was observed in the high-risk group in multiple cohorts.

### Functional analyses of the 9-gene signature

**FIGURE 6:**
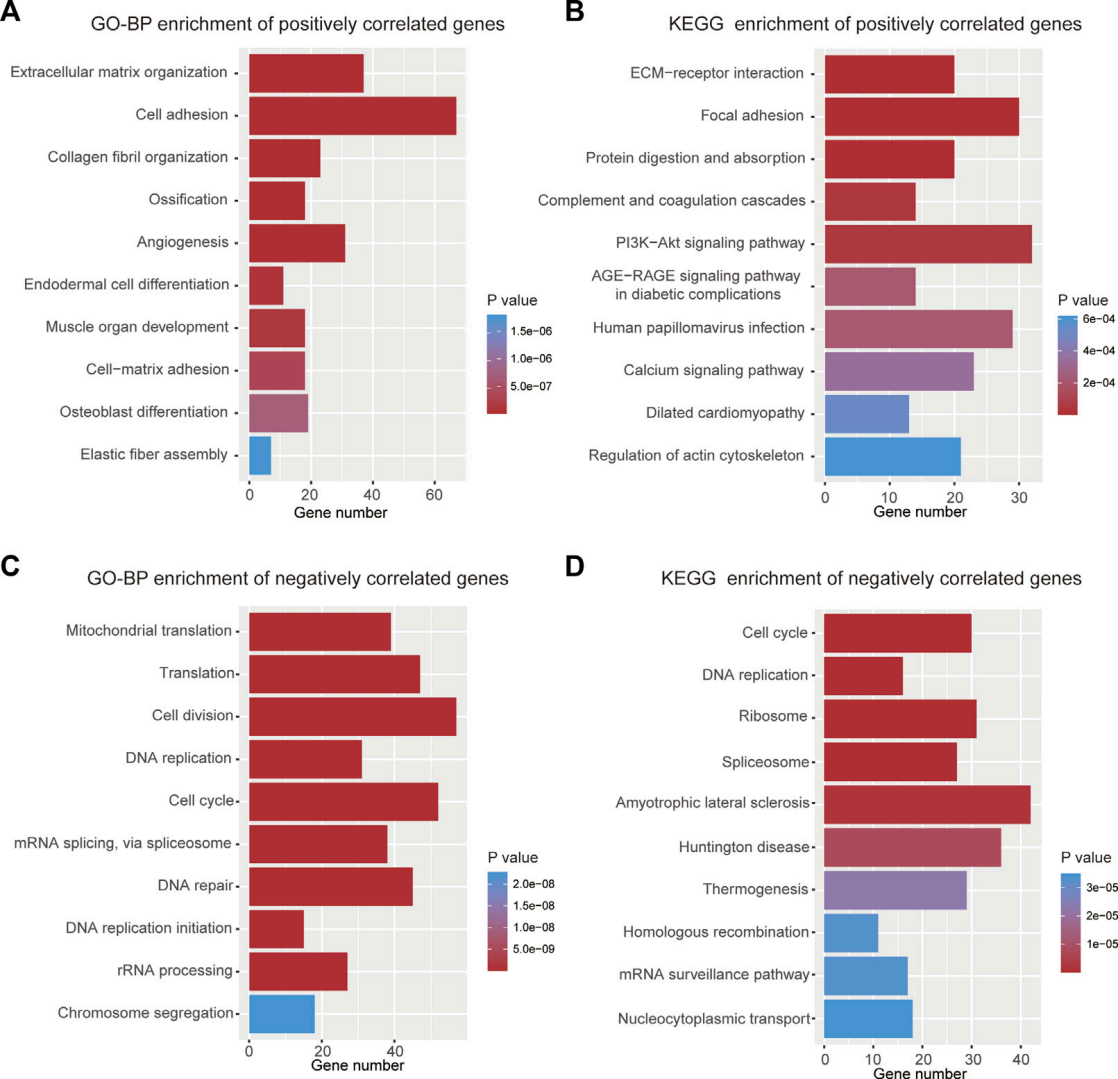
Functional analyses of the 9-gene signature. **(A)** Top 10 terms of GO-BP analysis for genes with positive correlation. **(B)** Top 10 terms of KEGG analysis for genes with positive correlation. **(C)** Top 10 terms of GO-BP analysis for genes with negative correlation. **(D)** Top 10 terms of KEGG analysis for genes with negative correlation.

To elucidate why high-risk scores lead to poor prognosis, biological process and KEGG enrichment analyses were performed on the top 1000 genes that were positively or negatively correlated with risk scores, respectively. In the biological process analyses, genes with positive correlations were found to be related to extracellular matrix organization, cell adhesion, and angiogenesis (Figure 6A), while negatively correlated genes were primarily associated with cell division, DNA repair, and cell cycle (Figure 6C). In the pathway enrichment analyses, genes with positive correlation were principally involved in ECM-receptor interaction, focal adhesion, and PI3K-Akt signaling pathways (Figure 6B), whereas genes with negative correlation were mainly enriched in the cell cycle and DNA replication (Figure 6D).

### Expression profile of the nine genes in TCGA cohort

**FIGURE 7:**
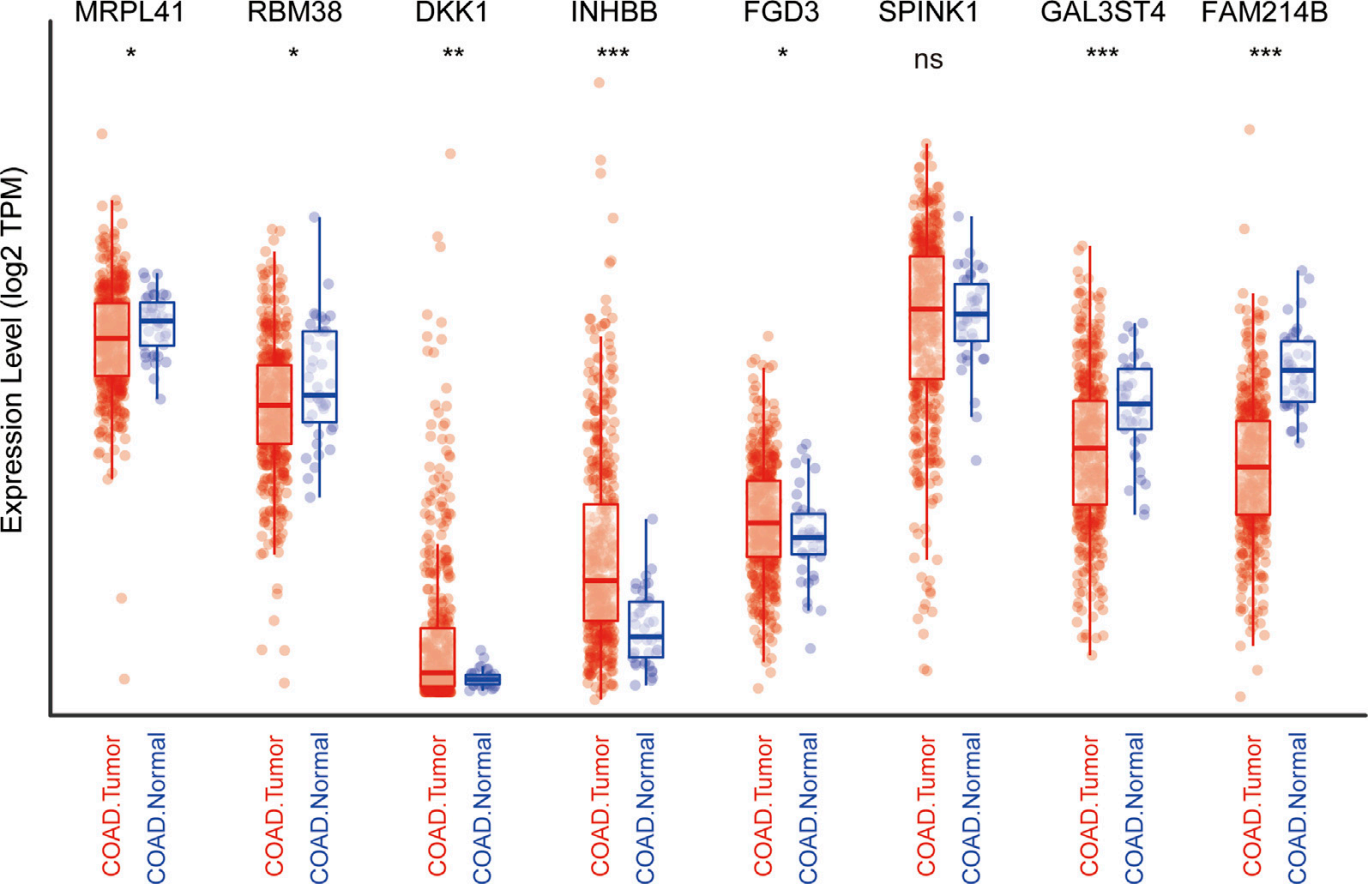
Expression profile of the nine genes in TCGA cohort. *p < .05, **p < .01, ***p < .001, ns: not significant.

We subsequently explored the differences in the expression of nine genes between normal and CRC tissues by analyzing TCGA data using the TIMER website (Li et al., 2016). Information about CTB-113P19.1. is not available on the website. As protective prognostic genes, MRPL41 and RBM38 were significantly downregulated, while FGD3 was upregulated and SPINK1 was not differentially expressed in CRC tissues. Among the risk prognostic genes, DKK1 and INHBB were markedly upregulated, while GAL3ST4 and FAM214B were markedly downregulated in CRC tissues (Figure 7).

### Experimental validation of MRPL41 and RBM38 expression patterns

**FIGURE 8:**
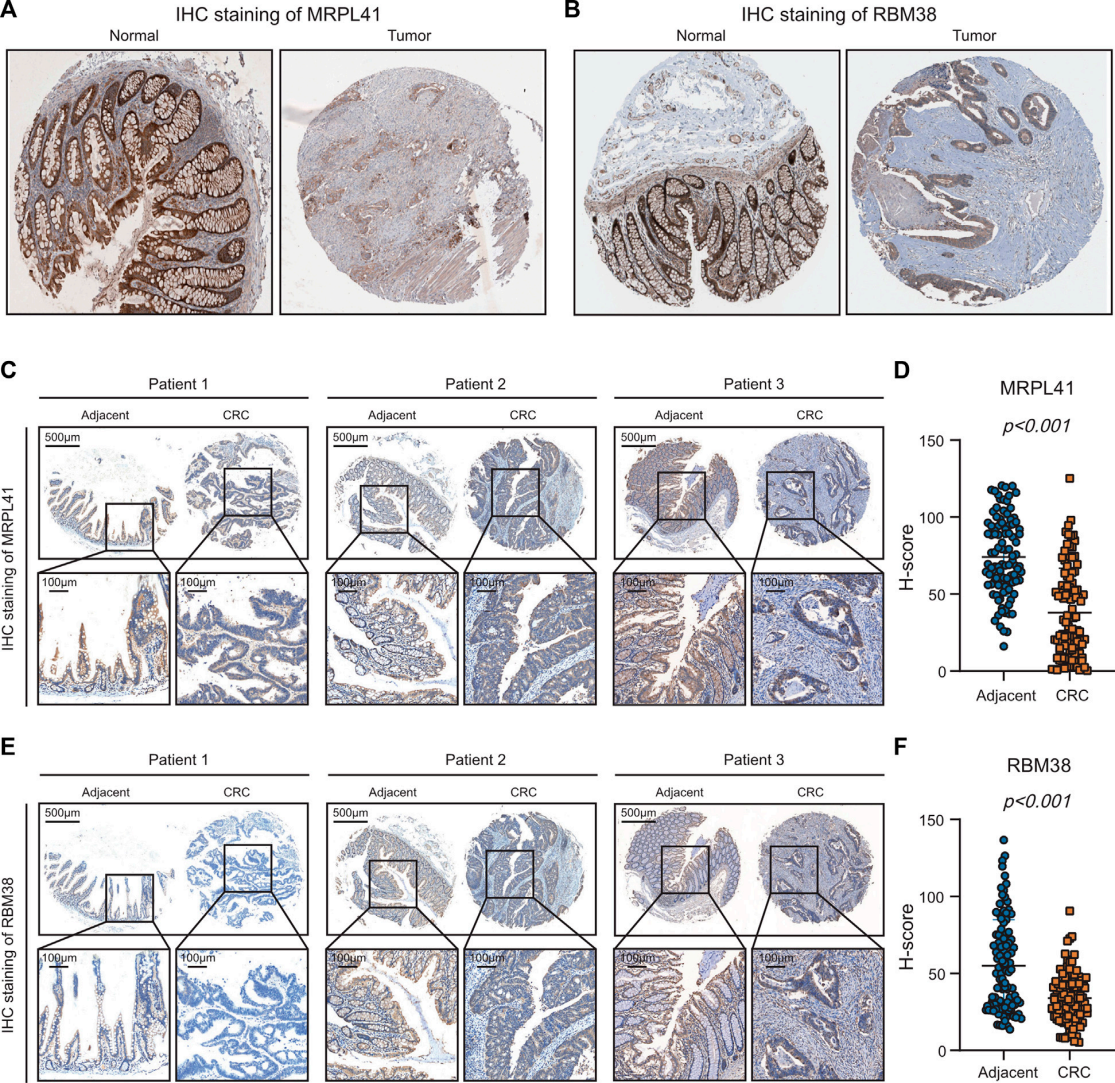
Experimental validations of MRPL41 and RBM38 expression patterns. **(A)** Representative IHC staining images of MRPL41 retrieved from the HPA database. **(B)** Representative IHC staining images of RBM38 downloaded from the HPA database. **(C)** Representative IHC staining images of MRPL41. **(D)** The quantitative H-scores of MRPL41 staining. **(E)** Representative IHC staining images of RBM38. **(F)** The quantitative H-scores of RBM38 staining.

From the above analyses of public RNA-sequencing data, we discovered that mRNA expression levels of MRPL41 and RBM38 were significantly decreased, whereas mRNA expression levels of DKK1 and INHBB were markedly elevated in CRC tissues. Protein expression levels of DKK1 and INHBB were also increased in CRC patients according to the literature (Sui et al., 2021; Zhou et al., 2022), but protein expression patterns of MRPL41 and RBM38 have rarely been reported. By analyzing the results of IHC staining assays from the Human Protein Atlas database (Uhlén et al., 2015), we found that protein expression levels of MRPL41 and RBM38 were dramatically decreased in CRC tissues (Figures 8A, B). The IHC assay further validated the downregulation of MRPL41 and RBM38 in CRC samples from our center (Figures 8C–F). These findings indicated that MRPL41 and RBM38 may play suppressive roles in CRC progression.

## Discussion

The high rates of postoperative recurrence and mortality after recurrence emphasize the importance of improving individual recurrence risk prediction for patients with stage II/III CRC (Ju et al., 2019). To date, multiple efforts have been made to develop reliable predictors of postoperative recurrence in CRC (Wang et al., 2015; Yang et al., 2020; Zhang et al., 2020). However, CRC features high degrees of genomic and transcriptional heterogeneity, and the suitability of current biomarkers for individual patients remains debatable (Kyrochristos and Roukos, 2019). As cancer treatment enters the area of precision medicine, efficacious recurrence assessment that considers genetic and genomic features is of great importance to guide clinicians in individualized follow-up and therapeutic strategies.

Accumulated evidence has proved that prognostic gene signatures have vast capabilities to aid clinical decision-making (Yu et al., 2019; James et al., 2022; Oliveira et al., 2022). We constructed and verified a prognostic 9-gene signature to predict the response of stage II/III CRC. In multiple cohorts, the risk model exhibited significant predictive performance. Univariate Cox regression analyses, together with the Venn diagram, initially screened 347 credible genes with the capacity to predict RFS. A 9-gene signature was developed using these genes. Survival analysis demonstrated the impressive predictive ability of this risk model. ROC curves together with time-dependent AUC values confirmed that our model was superior to previous models and clinical predictors in terms of predicting recurrence.

We then investigated the immunological correlation and biological function of the 9-gene signature. The results showed that the 9-gene signature was negatively associated with the infiltrative values of CD8^+^ T cell while positively correlated with those of regulatory T cell. Furthermore, high risk score was indicative of decreased GZMB expression and elevated HAVCR2 expression. These findings suggested that the 9-gene signature might be able to predict the response of stage II/III CRC patients to immunotherapy. To clarify the mechanism of gene signature’s effect on cellular immunology, we performed function analyses of genes correlated with this signature. Multiple signaling pathways related to the gene signature played fundamental roles in immune cell function. For example, overexpression of calcium-permeable channels at various locations of T cells is necessary for T cell activation, maturation and secretion of cytokines (Trebak and Kinet, 2019; Vaeth et al., 2020). Moreover, the PI3K-AKT pathway have long been considered to play an important role in the regulation of immune cell metabolism, growth, or survival (O’Donnell et al., 2018; Weichhart and Säemann, 2008; Hand et al., 2010). Above findings might partly explain how this signature influences the infiltration of immune cells and expression of immune checkpoint genes.

Among these nine genes, RBM38, SPINK1, DKK1, and INHBB are implicated in CRC tumorigenesis. RBM38 is downregulated in CRC cell lines and inhibits colorectal cancer cell growth and stemness by competitively binding to PTEN (Guan et al., 2021). However, the function of SPINK1 in CRC development remains unclear. Several studies have reported that SPINK1 is overexpressed in CRC and contributes to cell proliferation, migration, and invasion (Gouyer et al., 2008; Ida et al., 2015; Tiwari et al., 2015; Chen et al., 2022). However, some studies have found that SPINK1 is downregulated in CRC and indicates a favorable prognosis (Koskensalo et al., 2012; Koskensalo et al., 2013; Chen et al., 2016). Moreover, SPINK1 can reduce cetuximab resistance in CRC cells by effectively preventing PRSS1 from cleaving cetuximab (Tan et al., 2020). DKK1 is a well-known oncogene that fosters CRC cell growth, metastasis, chemotherapy resistance, and immune evasion (Qi et al., 2021; Sui et al., 2021; Zhao et al., 2021). INHBB is an unfavorable prognostic biomarker for CRC (Yuan et al., 2020). However, the specific biological functions of MRPL41, FGD3, GAL3ST4, CTB-113P19.1, and FAM214B in CRC remain largely unknown.

Accurate prediction of recurrence risk can help determine the applicability of adjuvant therapy, reduce overtreatment-related adverse effects, and avoid unnecessary medical expenses (Chan et al., 2021). The suitability of gene expression profiles for identifying patients with a high risk of recurrence has been proven in human cancers. The U.S. Food and Drug Administration (FDA) has approved a successfully developed genetic test called MammaPrint to evaluate the recurrence risk in stage I/II breast cancer patients (van ’t Veer et al., 2002). Our study is the first to establish a recurrence predictive signature for stage II/III CRC patients *via* credible RFS-related genes. Validation in five independent public cohorts, including American and French populations, enhances clinical compatibility. We hope to translate this gene signature into a commercial kit for easy clinical application.

Based on the retrospective data, the current study has several limitations. First, all cohorts used for survival analyses had a relatively small sample size, and the prognostic performance of the 9-gene signature needs larger cohorts and prospective studies to confirm the results. Second, patient treatment data were not available for several cohorts. The individual therapeutic benefits for patients in different risk groups are unclear. Third, the biological function and clinical relevance of the five genes in this signature remain largely unknown, and further *in vivo* and *in vitro* experiments are required.

In conclusion, we proposed a recurrence prediction model for patients with stage II/III CRC and comprehensively assessed the predictive capacity of this model. Therefore, it is desirable to guide personalized treatment and prolong patient survival.

## Data Availability

The datasets presented in this study can be found in online repositories. The names of the repository/repositories and accession number(s) can be found in the article/Supplementary Material.

## Abbreviations

AUC, Area under the curve; CRC, Colorectal cancer; EMT, Epithelial-mesenchymal transition; GEO, Gene Expression Omnibus; GO-BP, Gene ontology-biological process; GSVA, Gene set variant analysis; IHC, Immunohistochemical; KEGG, Kyoto Encyclopedia of Genes and Genomes; K-M, Kaplan-Meier; LASSO, Least absolute shrinkage and selection operator; ROC, Receiver operating characteristic; RFS, Recurrence-free survival; ssGSEA, Single-sample gene set enrichment analysis; TCGA, The cancer genome atlas; TNM, Tumor, node, metastasis.
