# Supplementary material for: A novel 9-gene signature for the prediction of postoperative recurrence in stage II/III colorectal cancer
Source: Front Genet. 2023 Jan 10;13:1097234. doi: 10.3389/fgene.2022.1097234 (PMC9871489; doi:10.3389/fgene.2022.1097234)
Supplement: Supplementary file 1 [file DataSheet1.docx]

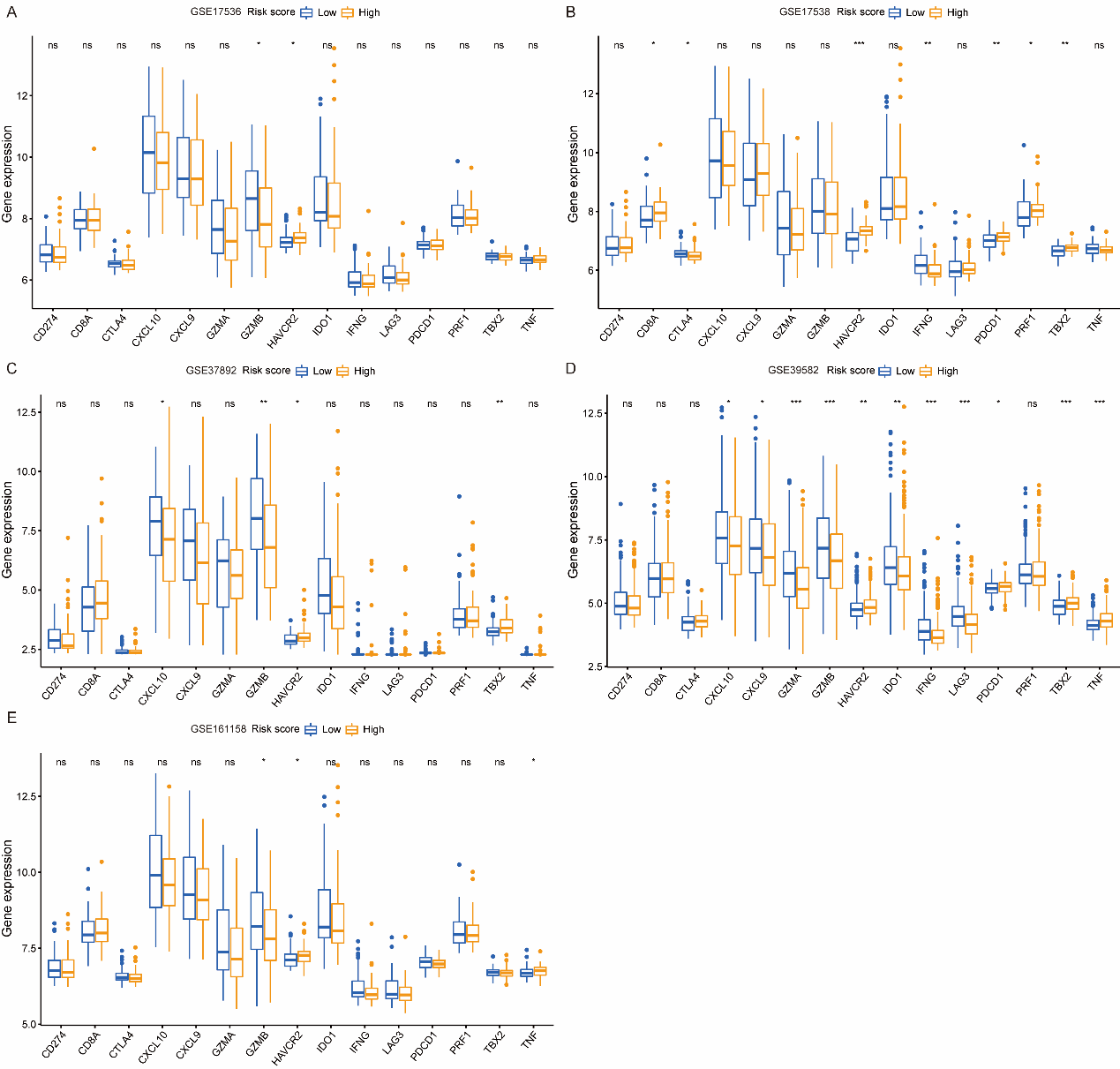


**Supplementary figure. 1** Expression of immune checkpoint genes in low-risk and high-risk groups in GSE17536 cohort **(A)**, GSE17538 cohort **(B)**, GSE37892 cohort **(C),** GSE39582 cohort **(D)** and GSE161158 cohort **(E)**. *P < 0.05, **P < 0.01, ***P < 0.001, ns: not significant.
